# Confronting Europe’s toxics trade from below: the contested global legacy of the 1976 Seveso disaster

**DOI:** 10.1080/13507486.2025.2494557

**Published:** 2025-07-09

**Authors:** Koen van Zon

**Affiliations:** Centre for Parliamentary History, Radboud University Nijmegen, Nijmegen, Netherlands

**Keywords:** Social history, European integration, environmental justice, hazardous waste, environmental governance

## Abstract

The 1976 Seveso disaster and its scandal-ridden aftermath had a lasting legacy that extended to the European Community (EC) and even beyond. When hazardous waste from the Seveso disaster site went missing, it generated a pan-European media scandal and a powerful symbol of the risks that modern industrialized capitalism produced. The scandal also revealed the perverse mechanisms that facilitated and even encouraged the dumping of chemical waste in and outside Europe. A variety of societal groups, ranging from consumers and environmentalists to development organizations, mobilized jointly to campaign against the trade in hazardous wastes. They all shared the same analysis, namely that the EC was both the core of the problem and the beginning of a solution. Setting out three case studies of grassroots mobilization in response to the trade in hazardous waste, this article examines how these activists found their way to EC institutions and how their campaigns evolved in the process. The first deals with the waste trade on the Common Market, the second with the global impact of waste exports from the EC and the third with a local anti-pollution movement emerging in response to the waste trade. Each of these mobilizations involved coalition building, allowing for a broad idea of environmental justice to emerge and for these coalitions to channel grassroots grievances with the waste trade to the EC institutions. In doing so, these grassroots mobilizations contributed to the EC becoming an increasingly important venue for global environmental governance towards the late 1980s.

## Introduction

At midday on 10 July 1976, residents in and near the Italian town of Seveso were shaken by a violent explosion at the local chemical plant. An error in the production process of 2,4,5-trichlorophenol, a substance used in the production of pesticides, caused the violent release of a cloud of highly toxic dioxin. After this cloud had descended over neighbouring towns just outside Milan, its devastating effects on the environment and residents became clear. Large numbers of animals died and children especially contracted a severe skin condition called chloracne. In the longer term, the number of people diagnosed with toxicity-related diseases such as cancer rose sharply, as did the number of women preventively aborting pregnancies. Yet, the impact of the Seveso disaster extended far beyond the Milan region.

Seven years later, in March 1983, news broke that waste from the disaster site that was contaminated with highly toxic dioxin had gone missing, after having been packed into 41 barrels and transported out of Italy. The waste going astray caused a pan-European scandal, mobilizing concerned citizens and organizations across Western Europe.^1^ It alerted Europeans to chemical hazards and the pervasiveness of the irregular as well as the sanctioned dumping of chemical waste. Across Europe and beyond, unprecedented numbers of people mobilized against (rumours of) such practices. Beyond targeting the responsible chemical corporations and national authorities, many of these mobilizations shared one striking characteristic: they also targeted the European Community (EC).

The growing salience of the trade in hazardous waste during the 1970s and 1980s ran parallel to the EC’s emergence as a global norm entrepreneur in that field.^2^ As the EC’s commitment to environmental governance grew, its effects became clear at the regional European level, but also locally and globally. As a response, the EC became the focal point of new forms of environmental activism at these three levels. This article charts this multilevel activism and how its development mirrored that of the EC.

Two perspectives have been dominant in scholarship on the question of hazardous waste, its disposal and trading. The first is a top-down perspective, which involves the economic dynamics of the development of the waste trade, and political and regulatory responses to this transnational and even global challenge. These histories play out at different levels, from the bilateral interaction between different national ‘waste regimes’ to the development of the global governance of hazardous waste.^3^ The second is a bottom-up perspective that focuses more on the waste trade’s effects on the ground as well as subsequent activist responses. These studies have shown how chemical risks were unevenly distributed and hit socially and economically vulnerable groups hardest – within countries and regions as well as globally – giving rise to the term ‘environmental justice’.^4^

In recent years, historians have combined the top-down and bottom-up perspectives, showing how local resistance against the dumping, transport or incineration of hazardous waste was part and parcel of a larger transnational movement that successfully pushed back against these practices.^5^ Where these studies tend to focus on the United States, this article underlines the importance of looking at the European context. Here, the EC and its unique character as a relatively young level of governance wedged between the national and the global allows for tracing what role it played in regulating the trade in hazardous waste and, in turn, how societal organizations responded to the role that the EC took up.

This article looks at the EC as a venue for societal mobilization against the hazardous waste trade along two axes.^6^ In a vertical sense, the EC’s multilevel system of governance makes it eminently suited to chart how societal mobilization against the waste trade played out at different levels and how regulatory responses at different levels were interconnected. Along this vertical axis, societal actors pursued a ‘politics of scale’, strategically addressing the political venues where they felt they could make a difference.^7^ Along a horizontal axis, societal actors formed ‘advocacy networks’ – voluntary and often ad hoc coalitions that reflected the dispersed impact that the hazardous waste trade had on public health, the environment and society at large.^8^

This article addresses the question of how grassroots societal actors navigated these axes, how their campaigns evolved as they developed in terms of composition, agenda and repertoire of action, and how this shaped the governance of the trade in hazardous waste in the EC. It does so by tracing the history of the contestation of Europe’s hazardous waste trade throughout three stages of its development – first at the European, then at the global and finally at the local level. It shows that the Seveso disaster cast a long shadow over each of these stages – with its lingering after-effects, impactful regulatory responses and the way in which societal actors across Europe and beyond used it as a cautionary tale against the trade in hazardous waste.

Archival research was conducted at the archives of EU institutions as well as at a number of archives which hold documents on (members of) organizations that were involved in campaigns against the hazardous waste trade, most notably the Greenpeace International Archive in the International Institute for Social History (IISH) in Amsterdam. Before delving into the three case studies of coalition activism, the first section traces the evolution of the trade in chemical wastes in the period preceding the 1983 Seveso waste scandal.

## The commodification and globalization of hazardous waste during the long 1970s

The chemical industry was a key driver behind Europe’s post-war economic boom. It created and met demand for a growing range of consumer goods, such as plastics, synthetic fibres like nylon, and the fertilizers and pesticides that had been instrumental to the so-called green revolution. Between the late 1940s and the late 1970s, the global production of chemicals expanded a staggering 900%.^9^ The ubiquity of chemicals went hand in hand with the problem of dumping, and the pervasive pollution of soil, surface and ground water and the dispersal of pollutants throughout the food chain.

In the late 1960s and early 1970s, the issue of chemical pollution and dumping emerged on the agendas of international organizations, including the 1972 United Nations (UN) Conference on the Human Environment in Stockholm. Surprisingly, however, it was the North Atlantic Treaty Organization (NATO) that led the way in exploring the problem and seeking to define what qualified as ‘hazardous waste’ and its due ‘disposal’. The directive the EC subsequently adopted on toxic and hazardous waste in 1978 largely built on the work done by NATO.^10^ It put in place a permit system for installations storing or handling hazardous waste and obliged member states to make plans for their disposal. The definition it laid down was quite broad and encompassed any waste that contained or was contaminated by a comprehensive list of substances and materials, which included pesticides, pharmaceuticals and various metal compounds, but excluded substances that were subject to different regulations, such as radioactive waste, explosives and effluents.^11^

This attempt at harmonization left member states ample latitude to handle hazardous wastes as they saw fit. Still, technical solutions such as incinerators and treatment plants were expensive and had a limited capacity. Under the ‘polluter pays principle’, which the directive reiterated, the due processing of hazardous waste thus became a more costly affair for producers and member states.^12^ Expanding the regulation on hazardous waste therefore had an unintended consequence: hazardous waste went from being a valueless by-product to a lucrative commodity. This gave rise to a lively international trade in hazardous waste, which had two faces.

Within Europe, the expansion of regulation, paired with the free movement of goods in the EC, meant that a European system for waste disposal emerged, whereby the suitable disposal site could be located outside the country wherein it was produced. By the mid-1980s, about 10% of the hazardous waste that was produced in the EC crossed borders, which amounted to two or three megatons annually.^13^ In member states of the Organization for Economic Cooperation and Development (OECD), this amounted to hazardous waste crossing a border once every five minutes.^14^ The trade between affluent and industrialized member states of the OECD made up the overwhelming majority of the global trade in hazardous waste.^15^

A much smaller, but much more notorious aspect of the global waste trade was that which flowed from Western Europe to other parts of the world. As the production of hazardous waste and its regulation expanded, discrepancies grew between jurisdictions, which allowed producers to offload their waste at a margin of the costs – often to low-income countries such as communist Eastern Europe and Africa, but also into international waters.^16^

Whereas these loopholes in the global patchwork of waste regimes provoked a race to the bottom dynamic, the actual scale of the trade in hazardous waste from the Global North to the Global South is subject to debate.^17^ There is an argument to be made that the figures we have of the waste trade in the 1970s and 1980s were only the tip of the iceberg, because much waste trading was clandestine, went under the radar, involved the mixing of hazardous waste with other products or their relabelling as other commodities. On the other hand, the most comprehensive survey of the global trade in hazardous waste that we have from the period was done by Greenpeace, which was prone to overreporting the problem.^18^ In the absence of conclusive data on the scale of the trade in hazardous waste, it was the global campaign against this trade that was key in increasing its salience, as this article will show.

Just as the Seveso disaster became a lasting symbol of chemical hazards, galvanizing societal and regulatory responses in Europe, the Love Canal scandal proved a catalyst in the United States. In 1978, it turned out that the Love Canal neighbourhood in Niagara Falls, New York, had been built on a toxic landfill, with catastrophic consequences for the environment and public health.^19^ ‘Love Canal’ was not just a local or national affair, however, as activists, media and third countries started pointing out. To address the question of hazardous waste exports, the United States developed a principle called Prior Informed Consent (PIC), which required importing authorities to consent to hazardous waste exports prior to shipment.^20^ PIC became a global norm, although the UN Environmental Programme (UNEP) and the OECD adopted it in much less stringent form.^21^

This emerging global system for governance the trade in hazardous waste framed the problem in economic terms – as an information asymmetry between the exporting and importing authorities on an otherwise legitimate market. In the EC, being a market project first and foremost, plans to regulate the trade in hazardous waste developed along the same lines.^22^ The European Commission had in fact already set to work on a directive on cross-border traffic in hazardous wastes when the news broke in 1983 that the Seveso dioxin had gone missing. Its preliminary proposal was to put in place a notification system, which required neither notification *prior* to the transport nor consent from the importing country.^23^ EC member states were free to adopt stricter measures than existing international agreements stipulated. France became one of these exceptions when it adopted a law in 1983 that allowed blocking exports when there were signs of hazards.^24^ By the early 1980s, the global governance of hazardous waste was thus a patchwork of regulations at different levels, riddled with loopholes. It often took scandals that exposed these loopholes for authorities to address them, such as the Seveso waste scandal did in the EC.

## The European dimension: the Seveso disaster and its pan-European aftermath

It was over six years after the Seveso disaster that rumours emerged that 41 barrels containing dioxin had left Italy. The waste from Seveso became a transnational concern overnight, as environmental organizations like Greenpeace started enquiring where it had gone.^25^ In Strasbourg, Members of the European Parliament (MEPs) called repeatedly upon the European Commission to find out where and under what conditions the barrels had been transported.^26^ It soon escalated into a pan-European media scandal with television crews and news reporters investigating rumours about the waste having been dumped in France, West Germany or elsewhere.^27^ The scare about dioxin and the uncertainty about its whereabouts fed into a narrative that ‘Seveso is everywhere’.^28^ In West Germany, where environmentalism was becoming an especially salient political theme, these rumours gave rise to local protests, where this slogan featured.^29^

Seveso thus became a powerful symbol of corporate irresponsibility and impunity. It united Europeans in a short-lived public sphere on hazardous waste. This transnational scandal was a platform for a campaign that started in France, but soon led to a European coalition of diverging organizations. They sought to retrieve the waste and hold its producer accountable, while exercising public pressure on the EC to address the hazardous waste trade. This section shows how the Seveso waste scandal galvanized the formation of a new kind of pan-European coalition of consumers and environmentalists, which managed to both mobilize consumers for a boycott and advocated EC legislative action.

In a turn of events frantically covered by European media, the barrels eventually turned up in an abandoned slaughterhouse in Northern France in May 1983. Scepticism and speculation about whether this was indeed the Seveso dioxin persisted, however, even after the contents of the barrels were incinerated in Basel in 1985.^30^ The Swiss multinational firm Hoffmann-La Roche was identified as the culprit in the scandal. One of its subsidiary firms, which ran the Seveso plant, had tasked a West German firm with the transport, which in turn had hired a French subcontractor. Executives at Hoffmann-La Roche refused to disclose any information about the deal on grounds of confidentiality, nor did they comment on the whereabouts of the barrels.

Social movements across Europe sought to retrieve the waste and call Hoffmann-La Roche to account. In April 1983, Greenpeace sought to alert Europeans to the scandal by symbolically blocking the access to offices of Hoffmann-La Roche in Basel and Paris with toxin barrels. Later that month, the Swiss Socialist Party briefly occupied the head office of Hoffmann-La Roche in Basel.^31^ The protest intensified when a coalition of organizations in France demanded Hoffmann-La Roche to either disclose the whereabouts of the 41 barrels or face a boycott across its product ranges, from pharmaceuticals (by doctors and pharmacies) to pesticides (by farmers) and cosmetics and perfumes (by consumers).^32^ The driving force behind this boycott was the French consumer organization Union Fédérale des Consommateurs/Que Choisir, as it joined forces with the French sections of the environmental organizations Friends of the Earth and Greenpeace.

The Seveso boycott soon attained a European character, spreading to West Germany, Italy, the Netherlands, Switzerland, Denmark and the United Kingdom, among other countries. It scaled up through the European associations that UFC/Que Choisir and Friends of the Earth were members of: the Bureau européen des unions de consommateurs (BEUC) and the European Environmental Bureau (EEB). These Brussels-based associations had been set up to represent the interests of member organizations vis-à-vis the EC. They functioned as platforms to build a constructive partnership with EC institutions and, to that end, to forge a European consensus among their member organizations.^33^ BEUC and EEB were therefore hardly in the habit of calling for consumer activism. Greenpeace, by contrast, was more geared towards direct action and hardly invested in lobbying national governments or EC institutions at this point in time.^34^ That is why this coalition worked: Greenpeace brought the campaigning zest for a single issue and expertise about the problem; BEUC and EEB brought the lobbying power – both in Brussels and through their member organizations.^35^

It was no coincidence that the Seveso boycott started with UFC/Que Choisir. Whereas consumer organizations in Northwest Europe conceived of their role primarily as independent channels for providing market information to members and as constructive partners for authorities, UFC/Que Choisir was a much more activist organization. It often organized grassroots mobilizations and sought confrontation with corporations, also on issues that extended beyond the classic repertoire of consumer organizations. In 1978, UFC/Que Choisir called for a boycott of Shell, after the sinking of the Amoco Cadiz tanker off the coast of Bretagne had caused a massive oil spill. UFC/Que Choisir again called for a boycott in 1980, this time of veal, after the discovery of baby food containing hormones that posed a risk to human health. Spearheading this boycott was UFC/Que Choisir’s director, François Lamy, which earned him the nickname ‘Lamy des consommateurs’.^36^ Lamy likened himself to the American consumer activist Ralph Nader, drawing inspiration from his campaigns and organizing.^37^ Lamy was a major factor in making this a European boycott – the first of its kind – after BEUC decided to amplify the call, to the dismay of some of its member organizations.^38^

With these boycotts, BEUC clearly diverged from its usual role as a constructive broker in European policymaking. This activist turn can be attributed in part to BEUC’s director, the Englishman Tony Venables. Having taken office in 1978, Venables wanted BEUC to exercise more political pressure through public campaigning, drawing inspiration from successful single-issue campaigns by organizations like Greenpeace.^39^ At the turn of the 1980s, moreover, the consumer boycott, specifically that against the food giant Nestlé, was a prime example of successful transnational campaigning. What had started off as an initiative by local activists from Minneapolis and New York due to Nestlé’s marketing practices of baby formula in the Global South became a global boycott, spearheaded by a growing coalition of transnational organizations and resulting in an international breast milk marketing code. This achievement attested to the idea that on a globalized market, regional mobilizations of consumers could call multinational corporations to account and impact regulation.^40^

Against this background, the Seveso boycott enjoyed broad support among BEUC member organizations. The Dutch Consumentenbond, for example, organized its own grassroots coalition of consumer groups, environmental organizations and, less successfully, doctors’ associations.^41^ Others were more pragmatic. The British Consumers’ Association, for example, only decided to join the boycott upon learning that its impact would be limited in the UK because Hoffmann-La Roche drugs were hardly available in pharmacies there.^42^ This broad support for the boycott is symptomatic of the shift that European consumer organizations underwent from the 1970s onward. In addition to focusing on material interests such as value for money and food safety, they also became advocates of broader post-material concerns such as environmental justice and quality of life.^43^ As the Consumentenbond stated in its magazine: ‘This is not just about Hoffmann-La Roche. It is about the consumer making clear to producers and authorities that he no longer tolerates environmental scandals.’^44^

In addition to consumer and environmental organizations, other groups soon started joining in the Seveso boycott. In some places, pharmacists put up signs or posters discouraging their customers from buying Hoffmann-La Roche products. Elsewhere, especially in West Germany, doctors stopped prescribing medicines by the company if alternatives were available.^45^ Distributing leaflets, sometimes supplied by Greenpeace, these medical practitioners also indicated which products to avoid and which alternatives were available.^46^ Generally speaking, however, doctors’ associations were quite reserved about joining the boycott, emphasizing that individual doctors were free to make their own choices.^47^

Different though the participating organizations may have been, it was precisely their diverging constituencies that made the coalition work. Through the boycott, Greenpeace and the EEB could speak on behalf of a new group in their ongoing fight against chemical pollution and dumping. Members of BEUC, meanwhile, could use the Seveso boycott to showcase their engagement with environmental issues.^48^ As much as environmental concerns prevailed in the campaign, the analysis that all participants made was really one of capitalism gone awry – from BEUC’s rather mild criticism of market failures and corporate accountability to Greenpeace’s more fundamental critique of industrial capitalism.^49^ The place of consumers in this constellation was ambiguous. Organizations like Greenpeace identified consumerism as a core problem of modern society, but also recognized consumers as a potential force for good, to be mobilized to hit corporations where it hurt: in their reputations and revenues. Indeed, the boycott certainly affected Hoffmann-La Roche’s image, as well as the value of its shares.^50^

In the end, the campaign ran only for one month. When the barrels turned up in Northern France, the campaign lost most of its urgency and symbolism. Although UFC/Que Choisir and Greenpeace kept pressing the Hoffmann-La Roche management to account for what had happened, Greenpeace campaigners realized by December that the Seveso boycott had ‘all gone cold in the public and [they, KvZ] should quietly bow out and save the boycott weapon for the future’.^51^ Brief though the boycott had been, it was enough to provoke a response from national authorities, corporations and the EC institutions.^52^

This European response came on top of what the EC institutions had already done prior to the 1983 waste scandal. The year 1982 saw the adoption of the so-called ‘Seveso directive’, which regulated industrial hazards. The European Parliament was especially attentive to Seveso. It set up the first committee of inquiry in its history on the subject, which concluded that the Commission had failed to adequately enforce the application of the 1978 directive on toxic and hazardous waste, and caused the Commission to ramp up its efforts in this regard.^53^ Further riding the wave of public sentiment following the waste scandal, the Parliament called upon the Commission for legislation on the waste trade.^54^ The West German liberal Martin Bangemann especially displayed a sense of pathos when he claimed that he had been ‘lucky to escape from a meeting with [his] life because of the Seveso waste question’, having been asked by citizens why the Commission and the Parliament had done so little to prevent the scandal or future scandals from happening.^55^

These public pressures compelled the Commission to go back to its 1982 draft proposal for a directive on the transboundary movement of hazardous waste and to take on board some of the Parliament’s demands. It was unwilling to abolish the trade in hazardous waste altogether, since it would compromise the principle of the free movement of goods. The Commission did concur with the Parliament’s demand for a PIC system, however, obliging the producer or handler of the waste to obtain consent from the receiving party prior to shipment.^56^ A proposal by the Parliament to make producers liable for any damages caused by the hazardous substances they produced was initially adopted by the Commission, but scrapped from the final directive by the Council of Ministers following an effective lobby by the chemical industry.^57^

The campaign that emerged in the wake of the Seveso waste scandal was unique, in that it mobilized consumers across the continent for an environmental cause, and fostered a hitherto unlikely alliance between organizations like BEUC and Greenpeace. The novelty and strength of this coalition lay not just in the fact that it connected Europe’s consumer and environmental movements horizontally, but also in its vertical layering, which meant that the grassroots boycott also translated into pressure on EC institutions. Although the retrieval of the Seveso dioxin stopped the coalition in its tracks, its campaign did convey a clear message to the EC that a tightening of proposals for regulating the hazardous waste trade was needed. As attention to chemical dumping grew, it became clear that Seveso was but the tip of the iceberg, and that the problem affected not just the Common Market, but that the Common Market itself was at the heart of the problem.

## The global dimension: the coalition for a ban on the hazardous waste trade

There was an irony at the heart of the 1984 EC directive on the transboundary movement of hazardous waste. Far from curtailing the waste trade in Europe, it served to mitigate the hazards that came with it. Just as tightening national environmental legislation had fostered a waste trade across the continent, tightening European legislation was a further incentive for producers to seek places where it cost them less to offload their waste. As a consequence of the 1984 directive, producers thus sought to ship their waste to less regulated parts of the world for processing or dumping, which could be up to 1000 times cheaper.^58^ It effectively legitimized an already existing problem, which investigative journalists and environmental organizations had drawn attention to since the early 1980s.^59^

This section shows how the Seveso coalition built on the momentum of their boycott campaign to put pressure on the EC to take up its global responsibility. Here too, grassroots pressures had a decisive influence on the campaign, which resulted in multilateral bans on hazardous waste exports in the late 1980s. Compared to the Seveso dioxin scandal, the globalization of the hazardous waste trade no longer concerned a highly mediatized scandal, but a more structural question of environmental justice. The EC’s role here was pivotal. Its rules on the waste trade did not extend beyond the Common Market, and therefore introduced double standards, as were also ingrained in the EC regulation of pesticides, chemicals and pharmaceuticals, which were associated with poisonings and deaths in the Global South.^60^

Hazardous waste exports became increasingly common during the 1980s. European states even stimulated them by offering large sums of ‘development aid’ to African states to take waste shipments. These deals often followed colonial patterns. In 1988, for example, France offered Benin 30 years of structural aid and a downpayment of 1.6 million dollars in return for taking radioactive and industrial waste exports. The deal was cancelled only after massive public outcry in France.^61^ In most cases, African states asserted their sovereignty to refuse such deals. Guinea-Bissau, for example, refused a 1988 deal to accept 15 million tonnes of toxic waste from corporations in return for US$600 million, which was twice its foreign debt.^62^

As the call to put an end to the global trade in hazardous waste became louder from the mid-1980s onward, the question of which authority was best suited to pursue this aim became poignant. Only UNEP had a global mandate, but many non-governmental organizations (NGOs) realized that it took the centres of the waste export, such as the EC, to take their responsibility. As the struggle against the waste trade went global, Brussels and Strasbourg therefore became crucial venues.

Leading the way in this lobbying effort was a coalition of organizations that built on the success of the Seveso boycott. It started with cooperation between BEUC and the International Organization of Consumers’ Unions (IOCU).^63^ These organizations were intertwined, since all BEUC members were also members of IOCU. Under the leadership of its Malaysian president Anwar Fazal (1978–84), IOCU underwent a shift from an organization which focused primarily on the concerns of consumers of the affluent West to an organization which had its centre of gravity in the Global South and focused much more on development, fundamental rights, basic needs and environmental health.^64^ Like his French colleague Lamy, Fazal drew inspiration from Ralph Nader’s campaigning zeal. Under Fazal’s leadership, IOCU set up several networks.^65^ Each of these networks coordinated the work of NGOs on a single issue, at a time when the number of international NGOs doubled.^66^

The networks that IOCU set up reflect the issues it prioritized. There was the Pesticide Action Network (PAN, 1982), for example, which in turn soon comprised 300 NGOs from around 50 countries. Health Action International (HAI, 1981), was a public health organization run from the IOCU office in The Hague by volunteers, which comprised some 50 organizations worldwide.^67^ Due to their decentralized and informal setup, these networks could quickly coalesce around a single issue and develop targeted campaigns, setting aside any other political differences that might exist between them.^68^

These network organizations, in turn, worked together. In 1985, BEUC, IOCU and its networks PAN, HAI, Seed Action Network and the International Coalition for Development Action, were joined by the EEB to form the Coalition Against Dangerous Exports (CADE). Much like the IOCU networks, it was a single-issue coalition, set up to address the double standards in the EC’s export regime. As a first initiative, CADE published a study by the journalist Andrew Chetley, entitled *Cleared for Export*, to raise awareness on the issue.^69^ Chetley had also played a key role in the Nestlé boycott, as director of the IOCU-affiliated Baby Food Action Network.^70^ In his report, Chetley drew attention to the consequences of the Common Market’s double standards in the areas of pharmaceuticals and hazardous waste regulation.^71^ Chetley and CADE frequently invoked the image of Seveso to make it relatable to their European audience. The 1984 Bhopal disaster in India stood out as an even more gruesome example of what would happen when European countries continued to externalize risks to the Global South: ‘The result will be more Bhopals, more Love Canals, more death, more unnecessary suffering.’^72^

The launch of the report was attended by several senior Commission officials and covered by news outlets across the Community. In its wake, CADE campaigners managed to arrange meetings with the relevant Commissioners and called upon all its member organizations to send a letter to Commission president Jacques Delors, calling to put an end to the double standards.^73^ There was considerable resistance to this aim. The EC business association, for example, forcefully objected to stricter export rules, arguing that they would introduce ‘barriers to a legitimate trade’. According to the corporate lobby, telling states in the Global South what they could and could not import amounted to paternalism, an argument that people inside the Commission employed as well.^74^

Nonetheless, there was growing momentum to regulate the export of hazardous waste. In 1986, the EC amended the 1984 directive on the transboundary movement of hazardous waste to extend the system of PIC to third countries.^75^ Furthermore, the Commission started work on a regulation to restrict the import and export of dangerous chemicals. In explaining its course of action, the Commission admitted that it had been sensitive to pressures from the public as well as civil society groups such as CADE.^76^

Meanwhile, the waste trade became an increasingly glaring global problem. As African states refused waste shipments, vessels carrying waste went adrift in search of a place to dock, sometimes for months on end. Branded toxic ghost ships, they became powerful symbols of the global waste trade, inciting unrest and protest wherever they went.^77^ Ship names like that of the Zanoobia, Karin B and Khian Sea frequently featured in news headlines. Against this backdrop, appeals by African leaders to stop the waste trade gained traction. After refusing hundreds of millions of dollars by US and French companies to accept waste exports, Gambia’s foreign minister Omar Sey called upon his fellow African leaders to do the same: ‘You liberate your countries from colonialism and you talk about imperialism, apartheid. But is that more horrible than dumping nuclear and toxic waste on an innocent country?’^78^ In a 1988 resolution, the Organization for African Unity called for a total ban on hazardous waste exports, characterizing the waste trade as ‘a crime against Africa and the African people’.^79^

The year 1988 was thus shaping up to become a decisive year for addressing Europe’s waste exports. UNEP had set in motion negotiations on a convention on the ‘transboundary movements of hazardous wastes and their disposal’, which would be concluded in Basel in 1989. The EC, meanwhile, had entered into negotiations with the African, Caribbean and Pacific Group of States (ACP) to negotiate the fourth generation of the Lomé Convention on trade and aid. Against this background, Greenpeace took its Toxics Campaign global. Tapping into its network in importing countries, Greenpeace sought to track the movements of toxic ghost ships and to create a stir around them, often to great effect.^80^ Whereas African states generally lacked a strong civil-society groundswell, NGOs like IOCU had committed to strengthening the consumer voice in Africa, especially on public health issues like the waste trade. To that end, IOCU organized a conference in June 1988, bringing together 18 consumer groups from African Anglophone countries in Nairobi, Kenya. The groups adopted a resolution condemning the export of hazardous waste as ‘toxic terrorism’, looking especially at UNEP for a solution.^81^

Negotiations on the Basel Convention, however, were moving in a more moderate direction that revolved around a system of PIC. IOCU, Greenpeace and the African Network of Environmental NGOs rejected this solution outright, however. Invited by UNEP director Mostafa Tolba to share their views, they argued that PIC would effectively ‘sanction and legitimize’ waste exports rather than restrict them, and that ‘nothing short of a global ban’ was acceptable.^82^ When it became clear that such a ban was off the cards, Greenpeace stated that it no longer supported the outcome of the Basel negotiations.^83^ Amplifying African grassroots grievances against the waste trade, IOCU and Greenpeace thus became advocates of banning the practice.

With several crucial international agreements under negotiation, CADE reconvened for a new campaign at the EC level, this time with Greenpeace as a member. In their new campaign, CADE called upon the EC to ban waste exports through the Basel and Lomé IV conventions.^84^ Although moderate organizations like BEUC were part of the coalition, CADE thus came to amplify grassroots calls from Africa. This position could count on support from a growing number of EC member states, but a Council initiative for a unilateral ban on toxic exports foundered due to a veto by the British government, which employed the paternalist argument that such a unilateral decision would amount to ‘commercial colonialism’.^85^

In 1989, the EC and the ACP states reached agreement on a multilateral ban on the export and import of hazardous and radioactive wastes in the framework of the Lomé IV negotiations.^86^ Aligning itself with the Global South, the EC took on a leading role in the Basel negotiations, even though it failed to ban the hazardous waste trade altogether. The United States Senate, moreover, refused to ratify the Convention. In protest of Basel’s shortcomings, 12 African states signed the Bamako Convention in 1991, unilaterally banning the import of hazardous waste from any country. Many states in the Global South followed this example. Whereas the trade in hazardous waste remained lively as ever, it arguably did halt the race to the bottom that flowed from North to South.^87^ Already by 1992, less than 1% of the hazardous waste produced in the United States and OECD-Europe was reportedly exported to the Global South.^88^

The Basel Convention marked the beginning of this trend and was the culmination of a longstanding struggle from below against a hazardous waste trade that can be traced back to the Seveso disaster. Some of the CADE member organizations continued their efforts, however.^89^ Greenpeace remained particularly active going into the 1990s, acting as a watchdog over member states’ compliance with EC regulations on hazardous waste transport and reporting violations to EC institutions.^90^ What set CADE apart from other organizations was that it focused exclusively on the EC. BEUC was at the heart of this coalition. It was the prime material contributor, and had strong global links through its association with IOCU, as well as strong links in Brussels through its contacts with policymakers and the EEB.^91^ It was not BEUC that set the agenda for CADE, however, but IOCU and Greenpeace, by amplifying African interests. As such, CADE became a strong advocate in Brussels for ending the double standards that were a direct result of the EC’s regulatory response to Seveso. The Basel Convention and Lomé IV agreement were thus culminations of a process in which the EC emerged as a norm entrepreneur in global environmental governance – spurred on by a broad coalition of different organizations.^92^

## The local dimension: the toxic boomerang of Deepsea Carrier

In March 1990, a group of women from the Apuglian town of Manfredonia, Italy, visited Strasbourg to join a meeting of the European Parliament’s Committee on the Environment, Public Health and Consumer Protection. They had travelled to Strasbourg to confront members of the Committee with the fact that the Italian government did not comply with European regulations with regard to a chemical plant in their area.^93^ Their history with pollution and chemical dumping dated back to around the time of the Seveso disaster, and came to a head when they were confronted with the fact that the global waste trade also affected their community. This section traces their mobilization against the waste trade as it scaled up to the European level. It shows how EC regulation of hazardous waste and industrial safety in the wake of the Seveso disaster became the object of local activists, who managed to navigate Europe’s multilevel environment even without aid from professional interest groups.

The origins of the protest date back to the late 1960s. Originally a fishing town, Manfredonia turned into a site for the production of fertilizers and caprolactam as part of a government plan to transfer industrial jobs from the North to the South of the country. As was typical for such moves southward, the state-controlled ENICHEM plant in Manfredonia faced safety issues from the beginning. This culminated in 1976 when an explosion at the plant sent a brown cloud over the city, followed by a snow of yellow slush. Thirty-two tons of arsenious dioxide rained down on Manfredonia that day, resulting in around 150 people being admitted to hospital with arsenic poisoning. Acting as if nothing had happened, the ENICHEM management sent employees back to work, ordering a team to clean up the site without any protection.^94^

The Manfredonia disaster happened just two months after that in Seveso. But whereas Seveso and its dioxin became pan-European symbols of the need to address chemical hazards, Manfredonia remained exposed to hazards that went largely unnoticed. In 1978, after an ammonia leak, the town was evacuated. In subsequent years, further leaks of ammonia, sulphur dioxide and nitrous gases followed. Public sentiments that had been building in the town came to a head in 1988, when the government directed a toxic ghost ship called the Deepsea Carrier to Manfredonia to have its cargo processed at the ENICHEM plant.^95^ A mass protest erupted, involving 40,000 locals, during which fishermen blocked access to the harbour, and protesters occupied the town hall, clashing with the police.^96^

The story of the Deepsea Carrier also had Italian origins. In 1987, two Italians made a deal with Sunday Nana, a Nigerian man who let out his backyard in the port town of Koko for storing 8000 barrels of waste, including highly toxic polychlorinated biphenyls (PCBs). When the barrels started leaking, plans were made to evacuate the town, but locals resisted. The Nigerian government sought to crack down on those involved and recalled its ambassador from Italy.^97^ It also made an appeal to the UN General Assembly to urge corporations to stop using Africa as a dumping ground.^98^ In July 1988, right around the time when negotiations over the Basel and Lomé IV conventions were in full swing, the vessels Karin B and Deepsea Carrier set out for Koko to retrieve the waste. In the following months, the ships were banned from docking in France, the Netherlands, Spain, the UK and West Germany, to overt criticism by the European Commission, until the Italian government decreed that the ships should return the waste to its country of origin.^99^ Greenpeace tracked the Deepsea Carrier on its voyage, with campaigners boarding it twice, sporting a banner that read ‘Stop Waste Trade’.^100^

The mass protests that the arrival of the Deepsea Carrier sparked in Manfredonia were connected to Seveso in more ways than one. For one thing, they gave expression to a deep-seated frustration: whereas Seveso in the affluent North had been in the centre of national and even European attention and had become a powerful symbol of chemical hazards, the plight of the citizens of Manfredonia was overlooked by authorities. Manfredonians saw the arrival of the Deepsea Carrier was an emblem of that neglect. Where the aftermath of the Seveso disaster ultimately drew attention to the issue of the waste trade, moreover, the Deepsea Carrier, a product of that waste trade, triggered protest against the industrial hazards that came with the presence of the ENICHEM plant – now partly subject to the EC Seveso directive.

The protests in Manfredonia united broad strata of local society. Involved in the protest committee were representatives of dockworkers, fishermen, different trade unions, environmental organizations (the World Wildlife Fund [WWF], Legambiente and the Italian Birdlife Protection League), healthcare workers, various political parties, women’s movements and ENICHEM workers. The latter group eventually separated, since the committee’s demand for closure of the ENICHEM plant went squarely against their interests as employees.^101^ The remaining groups organized themselves in the Citizen Committee for the Defence of Life, Health, Environment and Development – its name reflecting a broad appeal instead of a narrow environmentalist agenda. They set up a tent camp in Manfredonia’s main square, where members convened every evening for two years straight.

With time, and after the Deepsea Carrier had been diverted, the cross-movement character of the mobilization faded. Only the core group of the protest, the so-called Women’s Citizen Movement (Movimento Cittadino Donne), persisted. Among them were many teachers, as well as shopkeepers and housewives.^102^ They lodged an appeal against the Italian state, claiming that they had been denied freedom of information on ENICHEM’s impact on the environment and public health.^103^ Whereas local alliances became weaker, the Women’s Citizen Movement became embedded in a national network of local groups, often run by women, who also campaigned against industrial chemical hazards – occasionally together. Among these contacts were also women from Seveso.^104^

The Manfredonia women were not primarily driven by environmental concerns, however, but inspired by the thought of second-wave feminists such as the Italian philosopher Luisa Muraro. They claimed that the ENICHEM plant threatened life in the area and that they, as women, were more suited to represent this life than any other group – an ecofeminist critique that also surfaced in antinuclear protests, for example.^105^ In a pamphlet, the women argued:

As women and as mothers, being producers of life, we do not recognize ourselves in a mere logic of profit capable of monetizing any risk […]. We believe that all together we must discover a ‘feminine’ attitude towards nature, ‘of care and respect’ and not of ‘robbery and conquest’.^106^

The protest was also a feminist emancipatory act in itself: witness the fact that the women spoke of their public meetings as a ‘university on the square’.^107^

The Women’s Citizen Movement went on to pursue a politics of scale. Having sought justice at the regional and national level without effect, they now sought to call the Italian authorities to account through European institutions. They built their case on the 1982 EC Seveso directive, which compelled authorities to provide information about the safety of industrial activity to residents.^108^ They had access to the EC through an MEP for the Italian Communist Party, Adriana Ceci. A qualified paediatrician and an advocate of women’s emancipation who also hailed from Apuglia, Ceci was elected to the European Parliament in 1989. She visited Manfredonia at the invitation of the Women’s Citizen Movement and subsequently invited the women to Strasbourg in 1990. Following the example of her party colleague Vera Squarcialupi, who had already called attention to Manfredonia and the application of the Seveso directive in Italy, Ceci addressed numerous parliamentary questions to the European Commission on the matter, arguing that the ENICHEM plant was in breach of European environmental regulations.^109^

Parliamentary questions like these were actually powerful instruments: witness the fact that questions in similar cases directly caused the Commission to start infringement procedures or draft reasoned opinions against the Italian state.^110^ The European Parliament thus functioned as an intermediary for local activists to force their respective states to comply with European law, and to alert public opinion at home to shortcomings of their respective governments.^111^ In 1991, the European Commission indeed took the view that the Italian state had failed to adequately implement the Seveso directive. After a legal back and forth in the years that followed, the Commission eventually took Italy to the European Court of Justice for non-compliance with the EC treaties and won.^112^

The Women’s Citizen Movement itself also pursued a European legal avenue, after the Italian court of appeal acquitted the ENICHEM board. The women appealed to the European Court of Human Rights on Article 8 of the European Convention of Human Rights, which guaranteed the right to private and family life.^113^ In 1998, the Council of Europe’s Court ruled in their favour. The cases before both European courts were thus major victories for the Manfredonia women, although they had no immediate effects on the ground, as the production of fertilizers at ENICHEM had already ceased. The case of Manfredonia shows that the legacy of the Seveso disaster was local as well as global. In Manfredonia, the trade in hazardous waste and industrial hazards coalesced, sparking a grassroots mobilization that found its way to the EC, just as earlier coalitions had done.

## Conclusion

The Seveso disaster and its aftermath caused a ripple effect that shaped the governance of hazardous waste in the EC and beyond. What started with grassroots responses to the dioxin barrels going astray in 1983 culminated in a unique coalition of Brussels-based civil society organizations which spurred the EC on to intervene in the trade in hazardous waste – in Europe and beyond. In that sense, the aftermath of the Seveso disaster is part and parcel of the emergence of the EC as a norm entrepreneur for global environmental governance towards the end of the 1980s.

Throughout all this, the legacy of Seveso was omnipresent – both as a symbol and as the series of events that exposed regulatory failures in industrial safety and waste trading. Within Europe, few other events had such a lasting transnational impact on the public imagination of what chemical hazards looked like. It thus became a rallying cry for the organizations and activist groups featured in this history, who could use Seveso as a cautionary tale in their protests. At the level of the EC, the immediate regulatory response to Seveso and its aftermath was very comprehensive, ranging from the 1982 ‘Seveso’ directive on major accident hazards, the 1984 directive on the transfrontier shipment of hazardous waste on the Common Market and its 1986 revision that extended this regime beyond the EC.

As much as the EC response to the Seveso waste scandal was an attempt to prevent such scandals from happening in the future, it proved too noncommittal and loophole-ridden to put an end to dubious trading practices. That is why societal responses to the waste trade, whether they emerged at the local, national or transnational level, gravitated towards EC institutions. This dynamic played out along two axes. Horizontally, diverging groups mobilized and coalesced, ranging from the environmental movement, development organizations, feminist groups and consumer organizations from the Global South, as well as the affluent EC.

Diverse though their backgrounds often were, these organizations could form coalitions around shared agendas that went beyond environmental concerns alone. They advocated for environmental justice in a very broad sense, encompassing public health, social justice, gender equality and democratic values. The strength of these coalitions was that they showed that EC environmental policy was not a mere question of market regulation – it had strong socio-economic implications that affected their members and other Europeans, as well as people outside the EC.

Bundling their respective platforms allowed the coalition partners to make powerful claims on behalf of their respective memberships, but also to voice that message at different venues. Horizontal coalition formation reinforced the capacity of these alliances to shift venues along the vertical axis, concentrating their efforts where they sought to make impact. As a result, Brussels became a key venue for global organizations like Greenpeace and IOCU, but also for local communities.

This development shows that the 1980s were not just a decade of growing interdependence in global environmental governance, but also of a broad range of civil society organizations co-opting the concerns and even repertoire of action of environmental organizations. It is illustrative in this respect that BEUC, habitually a staunch defender of the logic behind the Common Market, got involved in a pan-European boycott and became an advocate of a global ban on the trade in hazardous waste, spurred on by its more radical coalition partners.

The growing salience of the questions of hazardous waste and industrial safety was the result of a reciprocal dynamic between the various forms of societal mobilizations discussed in this article on the one hand, and the EC’s growing commitment to transnational environmental problems on the other. Concerning the latter, this article has shown that the EC was rather responsive to the claims of various societal actors, even if they called into question the logic behind the Common Market. This responsiveness was not mere benevolence on the part of EC institutions towards societal organizations. It allowed institutions like the European Parliament to claim a democratic platform for their actions, and to show that the EC was not a mere technocratic market authority, but a key venue for environmental politics.

The various forms of grassroots activism that eventually reached Brussels reflected and affirmed the EC’s growing relevance in the area of environmental governance during the 1980s, at different levels. Most conventional was the activism that called upon the EC as an environmental legislator, such as in the case of the Seveso directive and the 1984 directive on the waste trade. The activism that called upon the EC to take up a global role by extending its Common Market rules to the rest of the world cast the EC in the role as a global norm entrepreneur. Lastly, the grassroots activism in Manfredonia called upon the EC in its role as an enforcer of its own environmental law. At each of these levels, societal actors came to recognize and effectively address the EC in its different roles, mobilizing the legacy of the Seveso disaster to advocate for environmental justice in Europe and beyond.

## Notes

1. Uekötter and Kirchhelle, “Wie Seveso nach Deutschland Kam,” 317–34; and Centemeri, “The Seveso Disaster Legacy,” 195–211.

2. Kelemen, “Globalizing European Union Environmental Policy”; Meyer, “Who Should Pay for Pollution?”; and Zito, *Creating Environmental Policy in the European Union*.

3. Sohm, “‘Big Clean’”; Borowy, “Hazardous Waste”; Brownell, “Negotiating the New Economic Order of Waste”; and Müller, “Hidden Externalities.”

4. Bullard, *Dumping in Dixie*; Naguib Pellow, *Resisting Global Toxics*; and Szasz, *Ecopopulism*.

5. Müller, *The Toxic Ship*; and Fazzi, *Smoke on the Water*.

6. Mosley, “Common Ground.”

7. Swyngedouw, “Neither Global Nor Local.”

8. Keck and Sikkink, *Activists beyond Borders*.

9. Müller, “Rettet die Erde vor den Ökonomen?,” 355.

10. Borowy, “How NATO Influenced International Governance in Hazardous Waste.”

11. Official Journal of the European Community (OJ) No. L 84, *Council Directive of 20 March 1978 on Toxic and Dangerous Waste* (March 31, 1978): 43–8.

12. Zito, *Creating Environmental Policy in the European Union*, 126–32; and Meyer, “Who Should Pay for Pollution?”

13. Borowy, “Hazardous Waste,” 11; and G. Yerkey, “EC Nations May Try to Halt Dumping of Toxic Waste in One Another’s Backyard,” *The Christian Science Monitor*, June 9, 1983.

14. Wynne, *Risk Management and Hazardous Waste*, 28–31.

15. O’Neill, *Waste Trading among Rich Nations*.

16. Sohm, “‘Big Clean.’”

17. For a general discussion of this phenomenon, see: Clapp, *Toxic Exports*, 21–38.

18. Montgomery, “Reassessing the Waste Trade Crisis”; and Vallette and Spalding, *The International Trade in Wastes*.

19. Szasz, *Ecopopulism*.

20. Müller, *The Toxic Ship*, 91–110.

21. Micklitz, “EC Regulation of the Export of Dangerous Pharmaceuticals to Third World Countries,” 41.

22. Cf. OJ No. C 112, *Programme of Action on the Environment* (December 20, 1973): 32; and OJ No. L 84, *Council Directive on Toxic and Dangerous Waste (78/319/EC)* (March 31, 1978): 45.

23. Historical Archives of the European Commission (HAEC), COM(82)892, *Proposal for a Council Directive on the Supervision and Control of Transfrontier Shipment of Hazardous Wastes within the European Community* (December 8, 1982): Article 3.

24. Chetley, *Cleared for Export*, 56.

25. Internationaal Instituut voor Sociale Geschiedenis (IISG), Greenpeace Nederland (GPNL) 1169, *Seveso: Report of an Inquiry* (November 1982).

26. Historical Archives of the European Parliament (HAEP), *Motion for a resolution tabled by Mrs. Squarcialupi and others with request for urgent debate […] on the transport of dioxin from Italy to another country in Europe* (November 15, 1982); *Motion for a resolution tabled by Mrs. Squarcialupi and others on behalf of the Communist and Allies Group with request for topical and urgent debate […] on the ultimate destination of the dioxin from Seveso* (April 11, 1983).

27. See: Institut National de l’Audiovisuel (INA): “Seveso/Déchets à Roumazières,” *Limousin actualités* (March 29, 1983); *Les déchets de Seveso sont-ils passés à Pontailler sur Saône?* (April 5, 1983). Accessed August 13, 2022. http://ina.fr.

28. Cf. Szasz, *Ecopopulism*, 54–6; Uekötter and Kirchhelle, “Wie Seveso nach Deutschland Kam”; Groupe de Travail Seveso, *Seveso est partout*; Koch and Vahrenholt, *Seveso ist Überall*; and “Überall ist Seveso,” *Der Spiegel*, May 29, 1983. This slogan subsequently resurfaced in the wake of the Chernobyl disaster. See: Arndt, *Tschernobyl*, 5, 69.

29. Lange, “A Deal over Dirt”; and “Gif Seveso niet in Münchehagen,” *Trouw*, April 25, 1983, 13.

30. Uekötter and Kirchhelle, “Wie Seveso nach Deutschland Kam,” 323–4; and De Marchi, “Seveso.” “Verschweigen, Abwiegeln und Dementieren,” *Der Spiegel*, May 29, 1983, 27–30.

31. Cf. “Verdwijning van Seveso-gif begint internationaal schandaal te worden,” *De Volkskrant*, April 9, 1983; “Stad Seveso leeft nog steeds in de ban van dioxine-wolk,” *De Volkskrant*, April 13, 1983, 4; and “Müssen denn erst Menschen krepieren?,” *Der Spiegel*, April 17, 1983, 114–22.

32. Cf. IISG, GPNL 1169, *Hoffmann-La Roche Threatened by an International Boycott* (April 15, 1983). Cf. Archives nationales de France (ANF), Amis de la Terre (ADLT) 20050521/58, *Press statement by Union Fédérale des Consommateurs, Greenpeace, Amis de la Terre and Fédération française des sociétés de protection de la nature* (April 21, 1983).

33. On the background of BEUC and EEB, see: Van Zon, Schimmel and van de Grift, “Vom Marktbürger zum Konsumburger?”; and Meyer, “Greening Europe?”

34. Cf. van de Grift, Rodenburg, and Wieman, “Milieuactivisten in Maatpak”; “Verdwijning van Seveso-gif begint internationaal schandaal te worden,” *De Volkskrant*, 9 April, 1983, 7.

35. Zito, *Creating Environmental Policy in the European Union*, 132–5.

36. “The Friend of Consumers.” See: B. Leucht, A. Schimmel and K. van Zon, “Interview with Tony Venables,” *EU-Bouwers aan het Woord*. Accessed April 24, 2025. https://oralhistoryeu.nl.

37. M. Hunter, “François Lamy: How France’s Nader Won Ban on Hormone-Treated Meat,” *The Washington Post*, December 24, 1988.

38. See the forthcoming Ph.D. Thesis by Alessandra Schimmel (Utrecht University). Historical Archives of the European Union (HAEU), BEUC 130, *Letter from A.G. Fransen to Tony Venables* (2 October 1980).

39. *Interview with Tony Venables*.

40. Adler, *No Globalization without Representation*; and Sasson, “Milking the Third World?”

41. Cf. “Consumentenbond wil boycot van medicijnen Hoffmann-La Roche,” *Leeuwarder Courant*, 25 April (1983): 13; and “Boycot medicijnen nog zonder effect,” *Trouw*, April 26, 1983, 11.

42. See note 39 above.

43. See the forthcoming PhD Thesis by Alessandra Schimmel (Utrecht University).

44. IISG, GPNL 1169, “Vermijdt produkten van Hoffmann-La Roche,” *Consumentengids*, June 1983.

45. See: Schweizerisches Sozialarchiv, F 5107-Na-03-038-002, *Dioxin Skandal, Bellevue Apotheke, April 1983*. Accessed August 8, 2022. bild-video-ton.ch/bestand/objekt/Sozarch_F_5107-Na-03-038-002. “US National Library of Medicine, A025605,” *Boycottons Hoffmann-La Roche*. Accessed August 8, 2022. https://collections.nlm.nih.gov/catalog/nlm:nlmuid-101438054-img; and “Boycot Hoffmann-La Roche breidt zich uit,” *Het Vrije Volk*, April 19, 1983, 7.

46. Cf.: F. Derz, *Alternative zu Hoffmann-La Roche* (Gesundheitsladen Berlin, 1983); and IISG, GPNL 1169, *Boycot Hoffmann Laroche* (Amsterdam, 1983).

47. Cf. IISG, GPNL 1169, *Landelijke Huisartsen Vereniging* (April 27, 1983); and Ibid., *Letter from Koninklijke Nederlandse Maatschappij ter bevordering der Pharmacie to Consumentenbond* (May 2, 1983); “Stimmen zur Giftfässer-Affäre,” *Deutsches Ärzteblatt* 80, April 29, 1983, 17, 20.

48. BEUC first concerned itself with environmental questions in the early 1970s. See: HAEU, BEUC 5, *Éléments pour un programme de travail* (1972). Thanks to Alessandra Schimmel for the reference.

49. See: Van de Grift, “Representing European Society.”

50. See note 39 above.

51. ANF, ADLT, *Telex from François Lamy to Katia Kanas* (May 30, 1983). IISG, GPNL 1169, *Meeting of Greenpeace and Hoffmann La Roche* (December 5, 1983). *Joint Declaration Hoffmann-La-Roche and Greenpeace. Greenpeace ends Hoffmann Boycot* (December 7, 1983). *Internal Greenpeace Telex* (December 29, 1983).

52. “Oproep tot boycot van Laroche,” *NRC Handelsblad*, April 20, 1983, 13; and “Bayer staakt maken levensgevaarlijk PCB,” *De Volkskrant*, April 22, 1983, 1.

53. Meyer, “Pushing for a Greener Europe,” 72–7.

54. See: HAEP, *Report drawn up on behalf of the Committee on the Environment, Public Health and Consumer Protection on the proposal […] for a directive on the supervision and control of transfrontier shipment of hazardous wastes within the European Community. Rapporteur: Mrs Van Hemeldonck* (May 30, 1983): 22–3.

55. *Debates of the European Parliament* No. 1-297 (April 12, 1983): 49.

56. On the Commission’s response to the Parliament’s demands, see: *Debates of the European Parliament* No. 1-300 (June 8, 1983): 114–16.

57. Cf. HAEU, CM2 1984/566-8, *CEFIC Memorandum on the amended proposal for a Council Regulation (EC) on the supervision and control of transfrontier shipment of hazardous wastes within the European Community* (November 8, 1983) 3. OJ No. L 326, *Council Directive on the Supervision and Control within the European Community of the Transfrontier Shipment of Hazardous Waste (84/631/EC)* (December 13, 1984): 31–41.

58. IISG, GPI 5881, *Waste trade correspondence* (date unknown).

59. Müller, *The Toxic Ship*; Weir and Schapiro, *Circle of Poison*; and HAEU, BEUC 50, *Recommendations of Plenary session on Dumping of Hazardous Products, Technologies and Waste into Third World Countries* (June 23, 1981).

60. Chetley, *Cleared for Export*; and Weir and Schapiro, *Circle of Poison*.

61. Brownell, “Negotiating the New Economic Order of Waste.”

62. Montgomery, “Reassessing the Waste Trade Crisis,” 6; IISG, GPI 6187, D. Mpondah, “Africa Grapples with Toxic Dumping Problem from the West,” *Third World Network Features* (1988).

63. HAEU, BEUC 328, *Exports of Dangerous Medicinal Products from the Countries of the European Community to the Third World. BEUC and IOCU Recommendations for Community legislation* (Brussels, March 4, 1985).

64. Van de Grift, “Of Consumers and Conservers.”

65. Hilton, *Prosperity for All*.

66. Levsen and Patel, “Imagined Transnationalism?”

67. Health Action International, *Pills, Politics and Practice: 25 Years of Promoting People-Centred Medicines Policy* (Amsterdam, 2006). Accessed August 8, 2022. http://haiweb.org; and Pellow, *Resisting Global Toxics*, 77–8.

68. Hilton, *Prosperity for All*, 127; Adler, *No Globalization without Representation*, 67–71.

69. Chetley, *Cleared for Export*.

70. Adler, *No Globalization without Representation*, 60.

71. Chetley, *Cleared for Export*, 64–75.

72. Ibid., 43. HAEU, BEUC 328, *Special Report Drugs and Agrichemicals: Chemical Exports to the Third World* (March 1985).

73. Personal Archive of Wilbert Bannenberg, *Launch of ‘Cleared for Export’ and follow-up* (October 10, 1985).

74. Cf. The answer given by Commissioner Narjes. OJ No. C 245, *Written question no. 648/82 by Mrs. Poirier to the Commission of the European Communities* (20 September 1982) 10; and Y. Domzalski, “Need for European Controls,” in *Drugs and Agrichemicals* (ICDA: Brussels, 1985) 7. Cited in Chetley, *Cleared for Export*, 55; see also: Müller, “Hidden Externalities,” 67.

75. OJ No. L 181, *Council Directive of 12 June 1986 amending Directive 84/631/EC on the Supervision and Control within the European Community of the Transfrontier Shipment of Hazardous Waste (86/279/ EC)* (4 July 1986): 13–5.

76. HAEC, COM (85) 843, *Proposal for a Council Regulation Concerning Export From and Import into the Community of Certain Dangerous Chemicals. Explanatory Memorandum* (November 15, 1985).

77. Müller, *The Toxic Ship*.

78. IISG, GPI 6187, D. Mpondah, “Africa Grapples with Toxic Dumping.”

79. IISG, GPI 6183, *“The Road to Basel.” Statement by Dr. Mostafa K. Tolba. Executive director UNEP* (16 November 1988).

80. Müller, *The Toxic Ship*, 111–32.

81. Cf. IISG, GPI 6184, *Resolution by Participants of the IOCU Conference “Consumers in Africa – Meeting the Challenge”* (Nairobi, 14/18 June 1988); Hilton, *Prosperity for All*, 108–12.

82. Amsab-Institute of Social History (AMSAB-ISG), 293-525, *Letter from Jane Bloom and others to Mostafa Tolba* (October 28, 1988).

83. Cf. IISG, GPI 943, *Report on Basel Convention on Liability Protocol, attended by Kevin Stairs for Waste Trade* (July 2/6, 1990). IISG, GPI 5876, *Waste Trade Campaign – Political Strategy Meeting* (May 12, 1989).

84. HAEU, BEUC 328, *Environment, Development and Consumer Groups Call for a Ban on the Export of Hazardous Wastes. Statement by CADE* (August 31, 1988).

85. S. Islam and P. Smith, “Dirty Games in Brussels,” *South* (August 1988), printed in: Third World Network, *Toxic Terror*, 72.

86. OJ No. L 229, *Fourth ACP-EC Convention signed at Lomé on 15 December 1989* (17 August 1991): 3–220.

87. O’Neill, *Waste Trading among Rich Nations*, 40–9.

88. Borowy, “How NATO Influenced International Governance in Hazardous Waste,” 315–16.

89. See the work of the newly founded Basel Action Network and the International Toxic Waste Action Network.

90. See: HAEC, BAC 1995/554 322–342 and BAC 1995/554 401–405.

91. AMSAB-ISG, 457-588, *PAN – Vergadering* (Brussels, January 31, 1985).

92. Kelemen and Vogel, “Trading Places.”

93. Malavasi, *Manfredonia*, 132.

94. Barca, “Bread and Poison.”

95. Malavasi, “Ecological and Democratic Crises.”

96. Malavasi, *Manfredonia*.

97. Vallette and Spalding, *The International Trade in Wastes*, 334–7; and Third World Network, *Toxic Terror*, 8–9, 70.

98. See note 78 above.

99. J. Pienaar, “EC Commissioner Attacks Delays in Curbing Toxic Waste,” *The Independent*, November 29, 1988.

100. Vallette and Spalding, *The International Trade in Wastes*, 334–7.

101. Malavasi, *Ecological and Democratic Crises*.

102. Ibid.

103. European Court of Human Rights (ECHR), *Judgement 116/1996/735/932: Case of Guerra and others v. Italy* (19 February 1998).

104. Malavasi, *Manfredonia*, 114–15, 128–9.

105. Kirchhof and Meyer, “Revealing Risks,” 344–6.

106. Malavasi, *Manfredonia*, 232, translation by Koen van Zon.

107. Malavasi, “Ecological and Democratic Crises,” 167.

108. Cf. Malavasi, *Manfredonia*, 233–7; OJ L 230, *Council Directive 82/501/EC of 24 June 1982 on the Major-Accident Hazards of Certain Industrial Activities* (5 August 1982): 1–18.

109. OJ C 173/5, *Written Question No. 2079/83 by Mrs Vera Squarcialupi to the Commission of the European Communities. Subject: Compliance with the Directive on the Major-Accident Hazards of Certain Industrial Activities* (February 21, 1984); HAEU, CPPE 1867, V. Squarcialupi, “L’Italia è sempre ultima,” *L’Unita*, March 12 (1984). Debates of the European Parliament, *Oral Question No 121, by Mrs Ceci (H-935/90)* (October 10, 1990): 232–3.

110. Cf.: OJ No. C 295, *Written Question No 426/87 by Mrs Vera Squarcialupi to the Commission of the European Communities* (November 5, 1987): 29; European Court Reports, Case C-33/90, *Judgement of the Court of 13 December 1991 – Commission of the European Communities v Italian Republic* (1991): I-5988; OJ No. C 110, *Written Question No 738/88 by Mr Francois Roelants du Vivier to the Commission of the European Communities* (May 2, 1989): 11–12; and OJ No. C 233, *Written Question No 910/89 by Mr Gianfranco Amendola to the Commission of the European Communities* (September 17, 1990): 2.

111. Van Leeuwen and Van Zon, “Preparing for Disaster.”

112. European Court Reports, Case C-336/97, *Judgement of the Court of 17 June 1999. Commission of the European Communities v Italian Republic* (1999): I-03771.

113. ECHR, *Judgement 116/1996/735/932*.
